# The Chain Mediating Effect of Sleep Quality and Family Resilience Between Depressive Mood and Treatment Compliance in Continuous Blood Purification Patients

**DOI:** 10.62641/aep.v54i4.2183

**Published:** 2026-08-15

**Authors:** Wei Dong, Xin Zhao, Qiuxia Gao

**Affiliations:** ^1^Department of Hematology, First Affiliated Hospital of Heilongjiang University of Chinese Medicine, 150040 Harbin, Heilongjiang, China; ^2^Department of Nephrology, West China Hospital of Sichuan University, 610041 Chengdu, Sichuan, China; ^3^Renal Dialysis Unit, Beijing Anzhen Hospital, Capital Medical University, 100029 Beijing, China

**Keywords:** continuous blood purification, depressive mood, treatment compliance, sleep quality, family resilience, mediation analysis

## Abstract

**Background:**

Patients undergoing continuous blood purification (CBP) face considerable physical and psychological burdens, and the prevalence of depressive mood is high, which adversely affects treatment compliance. This study aimed to investigate the chain mediating roles of sleep quality and family resilience in the relationship between depressive mood and treatment compliance among patients with CBP.

**Methods:**

A cross-sectional study was conducted in 195 patients with CBP from May 2024 to May 2025. Assessment included the Self-Rating Depression Scale (SDS), Pittsburgh Sleep Quality Index (PSQI), Family Resilience Assessment Scale-Chinese version (FRAS-C), and Dialysis Treatment Adherence Scale (DTAS). We performed Pearson correlation, multiple linear regression, and bootstrap mediation analyses (PROCESS Macro Model 6) to test the hypothesized chain mediation model.

**Results:**

The mean scores were SDS 52.64 ± 5.82, PSQI 10.55 ± 4.08, FRAS-C 87.09 ± 11.24, and DTAS 70.95 ± 13.25. Depressive mood was significantly associated of poorer sleep quality (β = 0.521, *p* < 0.01) with lower family resilience (β = –0.391, *p* < 0.01), which in turn were associated with lower treatment adherence (β = –0.253 and β = 0.192, respectively, both *p* < 0.01). The total indirect effect accounted for 41.74% of the total effect, with the chain path (depression → sleep → resilience → adherence) contributing 5.06%. After adjusting for age, the model remained significant.

**Conclusions:**

Improving sleep quality and enhancing family resilience may be potential strategies to mitigate the adverse effects of depressive mood on self-reported treatment compliance in patients with CBP, though these findings require confirmation using adherence measures validated specifically for the CBP population.

## Introduction

Continuous blood purification (CBP) is a life-sustaining treatment widely used for patients with various critical conditions [1]. CBP utilises extracorporeal circulation technology to maintain microenvironmental homeostasis by continuously removing toxins and inflammatory mediators from the body [2]. At present, beyond nephrology, CBP plays an increasingly important role in non-kidney diseases [3]. CBP is delivered continuously at a slow rate, typically for 12 to 24 hours. This approach avoids sharp blood pressure fluctuations seen with traditional dialysis, efficiently eliminates excessive inflammatory mediators, and precisely regulates electrolyte and acid-base balance [3, 4]. CBP has demonstrated increasingly clinical value in patients with sepsis, acute pancreatitis and multiple organ failure [5, 6, 7]. However, the psychological burden associated with CBP should not be underestimated. Research indicates that patients receiving CBP often experience high levels of psychological distress [6]. Depressive mood severely affect patients’ quality of life and is closely associated with reduced treatment compliance [8]. Inadequate compliance can lead to suboptimal treatment efficacy, increased complications, higher hospitalization rates, and poorer survival [9, 10]. Thus, identifying key factors linking depressive mood and treatment compliance in patients with CBP is clinically crucial for improving outcomes.

Sleep quality is an important indicator of physical and mental health [11]. Sleep disorders are highly prevalent in haemodialysis patients [12]. Haemodialysis and CBP share similarities as renal replacement therapies involving blood purification processes, which may induce common physiological and psychological stressors such as fluid and electrolyte disturbances, treatment-related discomfort and long-term disease burden [13]. Existing research indicates that dialysis patients with poor sleep quality are more prone to emotional problems such as anxiety and depression, and have lower compliance with medication and dialysis schedules [14, 15]. Family resilience, defined as the adaptive capacity of the family system in response to stress and adversity, plays a unique and critical role in chronic disease recovery [16]. For patients with CBP, treatment imposes not only marked physical and psychological stress on the patients themselves but also substantial financial and caregiving burdens on their families. Patients with high family resilience are better able to integrate internal resources and receive emotional support, which helps them develop a positive attitude towards treatment and increase their confidence in coping with the disease [17].

Although existing studies have separately explored the relationships between sleep quality, family resilience, depressive mood and treatment compliance in patients with CBP, no study has systematically examined sleep quality and family resilience as sequential mediators between depressive mood and treatment compliance. Elucidating this indirect pathway will help reveal how depressive mood affects treatment compliance in patients with CBP and provide a theoretical basis for targeted clinical interventions. This study aimed to examine the mediating role of sleep quality and family resilience. The finding will deepen understanding of the complex relationship between depressive mood and treatment compliance in patients with CBP, inform targeted psychological and family support strategies in clinical practice, and ultimately improve treatment outcomes and quality of life, offering important theoretical and practical significance.

## Materials and Methods

### Patient Population

This was a cross-sectional study. We enrolled patients who received treatment in the nephrology department of West China Hospital of Sichuan University from May 2024 to May 2025. CBP treatment was provided by the hospital’s Extracorporeal Blood Purification Therapy Centre. All enrolled patients had acute kidney injury or acute-on-chronic renal failure requiring CBP, with indications including sepsis, acute pancreatitis, or multiple organ dysfunction. CBP was delivered via continuous veno-venous hemofiltration (CVVH) or continuous veno-venous hemodiafiltration (CVVHD), with each session lasting 12 to 24 hours, and the number of sessions varied according to clinical necessity. Patients on maintenance haemodialysis were excluded. All patients provided written informed consent form. The study protocol was approved by the Ethics Committee of West China Hospital of Sichuan University (Ethics Approval Number: 2024-105) and was conducted in accordance with the Declaration of Helsinki.

### Inclusion and Exclusion Criteria

The inclusion criteria were as follows: (1) age ≥18 years; (2) requiring CBP treatment; (3) conscious with normal communication and comprehension abilities; and (4) complete clinical data.

The exclusion criteria were as follows: (1) severe cardiovascular, cerebrovascular diseases, malignant tumours or other major diseases; (2) recent major negative life events; or (3) current participation in other psychological interventions or drug clinical trials.

### Data Collection Method

The investigation was conducted by three senior nurses who had undergone uniform training, and who conducted the face-to-face survey when patients were clinically stable and free from sedation, delirium, or obvious cognitive impairment during CBP treatment intervals. Prior to the commencement of the survey, the purpose, methodology and confidentiality of the study were explained in detail to patients, and questionnaires were distributed only after obtaining consent. We used unified instructions to facilitate patients understand the content of the questionnaires. For patients with poor eyesight or writing difficulties, the investigators read out the questions one by one and assisted them in filling in the questionnaires, ensuring a standardised and regulated survey process. The average time to complete all questionnaires was approximately 20–25 minutes per patient. All questionnaires were administered after CBP treatment, with the objective of ensuring that any discomfort experienced during the CBP treatment did not compromise the integrity of the survey results. Only clinically stable patients with clear consciousness and normal cognitive ability were included in the assessment to minimize the influence of sedation, delirium, or cognitive dysfunction on self-report outcomes. The collected questionnaires were immediately examined for completeness and validity on the spot, and invalid questionnaires were excluded from the study. A total of 210 questionnaires were distributed in this study, and 195 were recovered, with a valid recovery rate of 92.86%.

### Data Collection Content

We used the following standardized scales for data collection: Depressive symptoms were evaluated using the Self-Rating Depression Scale (SDS) [18]. SDS was developed by American psychiatrist Zung [18] in 1965 and translated into Chinese and introduced by Chinese scholars Wang and Chi [19]. SDS has 4 dimensions and 20 items, using a scoring scale of 1 to 4, with a total score ranging from 20 to 80 points. In this study, we used standardized scores (raw score × 1.25), ranging from 25 to 100 points, with higher scores indicating more severe depressive symptoms. In this study, the Cronbach’s α coefficient for this scale was 0.85.

Sleep Quality was evaluated using the Pittsburgh Sleep Quality Index (PSQI) [20]. The PSQI was developed by Buysse *et al*. [20] in 1989 and localized into Chinese by scholars Liu *et al*. [21]. The PSQI consists of 7 dimensions and 19 self-assessment items, with scores ranging from 0 to 21. Higher score indicate worse sleep quality. In this study, the Cronbach’s α coefficient for this scale was 0.81.

Family resilience was evaluated using the shortened Family Resilience Assessment Scale-Chinese version (FRAS-C) [22]. FRAS was developed by Sixbey [22] and localized and revised by Chinese scholars Li *et al*. [23]. FRAS-C encompasses three dimensions: utilizing social resources, maintaining positive perspectives, and family communication and problem-solving. FRAS-C consists of 32 items and uses a 4-point scoring system, with a score range of 32 to 128 points. Higher score indicate higher level of family resilience. In this study, the Cronbach’s αcoefficient for this scale was 0.78.

Treatment compliance was assessed using the Dialysis Treatment Adherence Scale (DTAS). DTAS is a scale specifically developed by Zhang and Huang [24] for evaluating dialysis patients with end-stage renal disease. Given that no validated treatment adherence scale currently exists for patients with CBP, we adopted the DTAS in this study for the following reasons: (1) CBP shares fundamental treatment principles with maintenance haemodialysis, including extracorporeal blood purification, vascular access maintenance, and anticoagulation requirements [25]; (2) the core dimensions of DTAS—medication adherence, dietary compliance, fluid restriction, and dialysis session attendance—are clinically relevant to patients with CBP, who require strict adherence to anticoagulation protocols, fluid management, and treatment duration (12–24 hours per session); (3) all CBP patients were conscious and communicative and able to understand and complete the self-report scale. Notably, the construct validity of DTAS for measuring adherence in acute/acute-on-chronic critically ill patients with CBP has not been formally established, which should be considered when interpreting the results. The DTAS consists of 4 dimensions and 23 items using a 5-point scoring system, with a total score ranging from 23 to 115 points. Higher scores indicate better treatment compliance. In our study, the scale showed acceptable internal consistency (Cronbach’s α = 0.82) in the CBP population, supporting its preliminary applicability in this context.

### Statistical Analysis

Statistical analysis was performed using SPSS 26.0 (IBM Corp., Armonk, NY, USA) software. Prior to parametric testing, we assessed the normality of continuous variables using the Shapiro–Wilk test. All variables demonstrated approximately normal distribution (*p* > 0.05), supporting the use of parametric statistical methods. Descriptive statistics summarized the demographic characteristics of the participants; measurement data are expressed as mean ± standard deviation (SD) and categorical data as counts (percentages). Differences in treatment compliance among patients subgroups were compared using independent samples *t*-test and one-way analysis of variance (ANOVA). Pearson correlation analysis explored relationships between depressive mood, sleep quality, family resilience, and treatment compliance. For categorical variables, age was dichotomized at 65 years, and the number of comorbidities was categorized into 1–3 or >3 based on clinical convention. A chain mediation model was constructed using PROCESS Macro for SPSS (Version 4.2, Model 6) to examine the mediating roles of sleep quality and family resilience. Multiple linear regression analysis was tested the paths in the model, with R^2^, adjusted R^2^, and F values reported to evaluate the model fit. The significance of regression coefficients was determined by *t*-tests and *p*-values. We used bootstrap resampling with 5000 samples to verify the significance of the mediating effects, generating bias-corrected 95% confidence intervals (CIs). If the 95% CI did not contain zero, the mediating effect was considered statistically significant. Age was included as a covariate in the model to adjust for its potential influence. All statistical tests were two-tailed, and a *p*-value < 0.05 was considered statistically significant.

## Results

### Scale Scores for Patients

This study included 195 patients treated with CBP. Mean SDS score was 52.64 ± 5.82, PSQI score was 10.55 ± 4.08, FRAS-C score was 87.09 ± 11.24, and DTAS score was 70.95 ± 13.25 (Table 1). The Cronbach’s α coefficients for each scale were 0.85, 0.81, 0.78, and 0.82, respectively, indicating good reliability of the scales.

**Table 1.** **Scale scores of the patients**.

Scale	n	Score range	Score	Cronbach’s α
SDS	195	25–100	52.64 ± 5.82	0.85
PSQI	195	0–21	10.55 ± 4.08	0.81
FRAS-C	195	32–128	87.09 ± 11.24	0.78
DTAS	195	23–115	70.95 ± 13.25	0.82

SDS, Self-Rating Depression Scale; PSQI, Pittsburgh Sleep Quality Index; FRAS-C, Family Resilience Assessment Scale-Chinese version; DTAS, Dialysis Treatment Adherence Scale.

### Basic Characteristics and Treatment Adherence

Patient basic characteristics and treatment adherence status were shown in Table 2. The DTAS score of patients ≤65 years was significantly higher than that of patients >65 years (73.55 vs. 69.06, *p *= 0.019, Table 2). Clinically, this suggests that younger patients may have better physical capacity and cognitive function to adhere to complex treatment regimens, whereas older patients may face additional barriers such as multiple comorbidities, frailty, or reduced social support. No significant differences were observed in treatment compliance based on sex, educational level, occupational status, marital status, place of residence and the number of comorbidities.

**Table 2.** **Patient characteristics and current status of treatment compliance**.

Variable		n (%)	DTAS (Mean ± SD)	Statistic	*p*
Age				*t* = 2.36	0.019
	≤65	82 (42.05)	73.55 ± 11.91		
	>65	113 (57.95)	69.06 ± 13.88		
Sex				*t* = –0.29	0.773
	Female	86 (44.10)	70.64 ± 13.64		
	Male	109 (55.90)	71.19 ± 12.98		
Education level				F = 0.06	0.942
	Primary school and below	77 (39.49)	71.00 ± 14.03		
	Junior school or senior high school	88 (45.13)	70.67 ± 13.21		
	College degree or above	30 (15.38)	71.63 ± 11.56		
Occupational status				*t* = 0.79	0.433
	Employed	113 (57.95)	71.58 ± 13.56		
	No occupation/retired	82 (42.05)	70.07 ± 12.83		
Marital status				*t* = 1.24	0.215
	Married	158 (81.03)	71.52 ± 13.10		
	Unmarried/divorced/widowed	37 (18.97)	68.51 ± 13.75		
Residence				*t* = –0.01	0.995
	Rural	105 (53.85)	70.94 ± 13.43		
	Urban	90 (46.15)	70.96 ± 13.10		
Comorbidities				*t* = 0.51	0.613
	1–3	78 (40.00)	71.54 ± 13.85		
	>3	117 (60.00)	70.56 ± 12.87		

DTAS, Dialysis Treatment Adherence Scale; SD, standard deviation.

### Correlation Analysis

SDS score was positively correlated between PSQI score (r = 0.520, *p* < 0.01), and negatively correlated between FRAS-C score (r = -0.547, *p* < 0.01) and DTAS score (r = -0.575, *p* < 0.01, Table 3). PSQI score was negatively correlated between FRAS-C score (r = -0.490, *p <* 0.01) and DTAS score (r = -0.523, *p* < 0.01). FRAS-C score was positively correlated with DTAS score (r = 0.504, *p* < 0.01). These correlation patterns suggest that patients with more severe depressive symptoms tend to have poorer sleep quality and lower family resilience, while better family resilience is associated of better sleep quality with higher treatment adherence, providing preliminary support for the hypothesized chain mediation model.

**Table 3.** **Correlation analysis**.

Variable	SDS	PSQI	FRAS-C	DTAS
SDS	1			
PSQI	0.520**	1		
FRAS-C	-0.547**	-0.490**	1	
DTAS	-0.575**	-0.523**	0.504**	1

SDS, Self-Rating Depression Scale; PSQI, Pittsburgh Sleep Quality Index; FRAS-C, Family Resilience Assessment Scale-Chinese version; DTAS, Dialysis Treatment Adherence Scale. **p < 0.01.

### Regression Analysis of the Chain Mediation Model

SDS score was significantly associated with PSQI score (β = 0.521, *p* < 0.01) explaining 27.1% of the variance (Table 4). SDS score and PSQI score showed significant associations with FRAS-C score (SDS: β = -0.391, *p* < 0.01; PSQI: β = -0.282, *p* < 0.01), with a joint explained variance of 35.9%. The direct association of SDS score with DTAS score was significant (β = -0.563, *p* < 0.01), explaining 33.5% of the variance. When PSQI score and FRAS-C score were added, the associations of these three variables with DTAS score remained significant (SDS: β = -0.328, *p* < 0.01; PSQI score: β = -0.253, *p* < 0.01; FRAS-C score: β = 0.192, *p* < 0.01), and their jointly explained variance increased to 42.8%.

**Table 4.** **Analysis of the chain mediation model**.

Equation		Overall fitting index			Significance		
Outcome	Prediction	R^2^	Adjusted R^2^	F value	β value	*t* value	*p* value
PSQI	SDS	0.271	0.263	35.636	0.521	8.31	<0.01
FRAS-C	SDS	0.359	0.349	35.717	-0.391	-5.693	<0.01
	PSQI				-0.282	-4.154	<0.01
DTAS	SDS	0.335	0.328	48.399	-0.563	-9.417	<0.01
DTAS	SDS	0.428	0.416	35.512	-0.328	-4.664	<0.01
	PSQI				-0.253	-3.775	<0.01
	FRAS-C				0.192	2.804	<0.01

SDS, Self-Rating Depression Scale; PSQI, Pittsburgh Sleep Quality Index; FRAS-C, Family Resilience Assessment Scale-Chinese version; DTAS, Dialysis Treatment Adherence Scale.

###  Effect Analysis of the Chain Mediation Model

The total observed association of SDS score with DTAS score was -1.284 (*p* < 0.01), of which the direct association was -0.748 (*p <* 0.01), accounting for 58.26% of the total observed effect (Table 5). Given the exploratory nature of the adherence measure in this CBP sample, these effect sizes should be interpreted with caution. The total indirect association was -0.536 (*p* < 0.01), accounting for 41.74% of the total association. Path-specific analysis showed that the indirect association of SDS score with DTAS score via PSQI score was -0.300 (*p* < 0.01), accounting for 23.36% of the total association. The indirect association via FRAS-C score was -0.171 (*p* < 0.01), accounting for 13.32% of the total association. The chain-mediated indirect association (depression → sleep quality → family resilience → treatment adherence) was -0.065 (*p* < 0.01), accounting for 5.06% of the total association.

**Table 5.** **The path and effect size of the chain mediation model**.

Effect	Path	Effect value	Boot SE	Boot 95% CI lower limit	Boot 95% CI upper limit	Proportion
Total effects		-1.284	0.136	-1.551	-1.016	100.00%
Direct effects		-0.748	0.16	-1.062	-0.433	58.26%
Mediated effects	Total mediated effects	-0.536	0.044	-0.712	-0.362	41.74%
	Path 1	-0.300	0.038	-0.414	-0.186	23.36%
	Path 2	-0.171	0.035	-0.263	-0.079	13.32%
	Path 3	-0.065	0.014	-0.108	-0.022	5.06%

Path 1: Depression → Sleep Quality → Treatment Adherence. Path 2: Depression → Family Resilience → Treatment Adherence. Path 3: Depression → Sleep Quality → Family Resilience → Treatment Adherence. SE, standard error; CI, confidence interval.

### Chain Mediation Model After Adjusting for Age

**Fig. 1.**
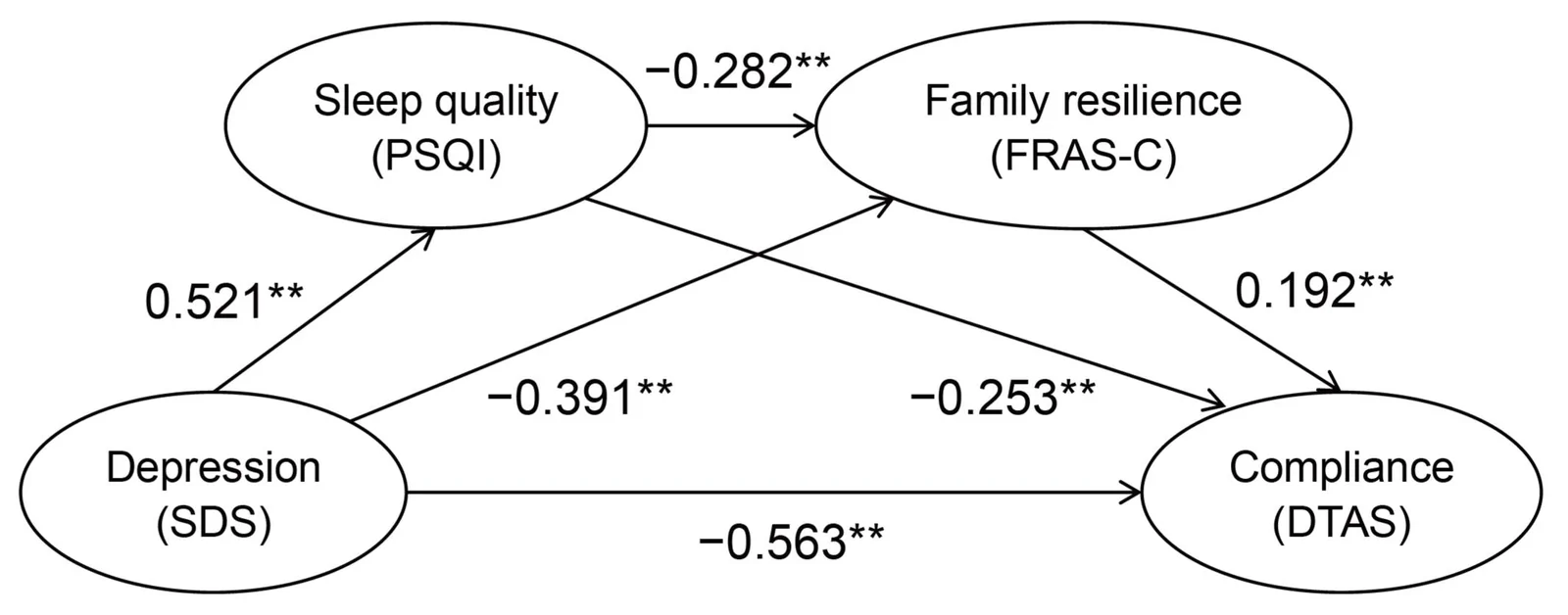
**Chain mediating effect model with standardized regression coefficients (β) from Table 4**. Values represent direct path coefficients from linear regression analyses, not mediated effect sizes. Overall indirect effects and effect decompositions are reported in Table 5. SDS, Self-Rating Depression Scale; PSQI, Pittsburgh Sleep Quality Index; FRAS-C, Family Resilience Assessment Scale-Chinese version; DTAS, Dialysis Treatment Adherence Scale. **p < 0.01.

In the chain mediation model analysis, age was adjusted as a covariate because univariate analysis identified it as the only demographic factor significantly associated with treatment compliance (Fig. 1). After adjustment, the path from SDS score to PSQI score, FRAS-C score, and DTAS score remained significant. This suggests that although age has some influence on treatment adherence, it does not change the pattern of associations among depressed mood, sleep quality and family resilience. This result further supports the reliability and stability of the findings.

## Discussion

This study systematically investigated the indirect associations of sleep quality and family resilience between depressive mood and treatment adherence in patients with CBP by constructing a chain mediation model. The findings suggest that depressive mood is associated with treatment adherence directly and indirectly through the sequential mediating pathway of sleep quality and family resilience, and that this pattern of associations remains robust after controlling for age. However, it is important to acknowledge that depressive mood, sleep quality, family resilience, and adherence-related behaviours may all be influenced by unmeasured factors such as illness severity, ICU-related experiences, treatment exposure, and cognitive status. Therefore, the present findings should be interpreted as exploratory, and the observed mediating effects should be considered hypothesis-generating rather than definitive.

From the results of the study, depressive mood was significantly and negatively correlated with treatment adherence in patients treated with CBP, consistent with findings from studies on patients receiving renal replacement therapies. Saguban *et al*. [26] reported in a cross-sectional survey of 668 haemodialysis patients that depression is a key factor affecting compliance, even among patients with high social support and self-efficacy. Similarly, Ling *et al*. [27] reported that depressive symptoms are the main factor affecting medication adherence in Chinese patients receiving renal replacement therapies. This may be related to the invasive nature and complication risks of CBP treatment, which can keep patients in a state of physical discomfort and psychological stress [28]. Depressive mood, a common psychological response, may reduce adherence to CBP frequency and dietary control by weakening the patient’s self-management motivation and reducing the perceived treatment benefit [29]. The research reports by Kaneez *et al*. [30] and Skoumalova *et al*. [31] confirm this conjecture: patients with higher levels of depression tend to have lower medication and dietary compliance. Additionally, neuroendocrine disturbances (e.g., hyperactivation of the hypothalamic–pituitary–adrenal axis) in depressive states may further exacerbate somatic complaints, creating a vicious cycle of “depression →somatic symptoms →reduced adherence” [32]. Notably, treatment adherence was significantly higher in patients ≤65 years than in older patients in this study, which may relate to a higher comorbidity burden , greater cognitive decline and functional impairment, and greater need for social support in older patients. However, age did not change the core association between depressive mood and adherence, suggesting that psychological intervention is necessary in patients of all ages. It is important to note that while evidence from haemodialysis populations provides a valuable reference, CBP differs substantially from conventional haemodialysis in several aspects. CBP is typically indicated for critically ill patients with conditions such as sepsis, acute pancreatitis, or multiple organ failure and is characterized by slower, continuous treatment over 12 to 24 hours, whereas haemodialysis is usually performed intermittently for patients with stable end-stage renal disease [33]. These differences may amplify the psychological and physical burden in patients with CBP, who often face acute life-threatening illnesses combined with prolonged treatment sessions. To date, studies specifically focusing on psychosocial factors in CBP populations remain limited. A study by Fan *et al*. [6] reported that patients with CBP in intensive care units experience significant psychological distress, aligning with our finding of high depressive symptom scores. However, unlike our study, their investigation did not specifically examine the potential mediating pathway between depressive mood and treatment adherence. The consistency of our findings with the broader literature on renal replacement therapies suggests that the negative effect of depression on adherence may be a common phenomenon across blood purification modalities; nevertheless, the greater clinical complexity of patients with CBP underscores the need for more tailored psychological interventions.

The mediating role of sleep quality reveals the interaction between physiological and psychological factors. Alshammari *et al*. [34] found in a study of 250 patients receiving renal replacement therapy that approximately one-third of them had poor sleep quality. Yazıcı and Güney [35] reported that 42.5% of patients receiving renal replacement therapies had sleep disorders, and the level of depression was independently associated with sleep quality. These studies indicate that sleep disorders are widespread among patients with CBP, consistent with the higher PSQI score in our study. We speculated that this may be closely related to the electrolyte imbalance, increased nocturnal urine output and psychological stress induced during CBP treatment. Mechanistically, depression may disrupt the sleep-wake cycle by activating the sympathetic nervous system and elevating cortisol levels [36]. Chronic sleep deprivation in turn enhances amygdala emotional reactivity and exacerbates depressive symptoms, creating a bidirectional effect [37]. Of course, this speculation requires verification through further animal experiments. At the same time, reduced sleep quality can directly affect patients’ daytime functioning, such as lack of concentration, making it difficult rigorously follow complex treatment regimens. This mediating pathway accounted for 23.36% of the total effect, suggesting that improving sleep quality may be a key target for breaking the “depression → low adherence” chain.

The mediating role of family resilience highlights the central value of social support systems in disease management. This study found that family resilience was positively associated with treatment adherence and that depressive mood indirectly affects adherence by reducing family resilience. For patients with CBP, treatment is a challenge not only for the individual but also for the entire family [38]. Highly resilient families can provide emotional support, financial security and information support, helping patients develop a positive attitude towards treatment and increasing their confidence in coping with the disease. Research by Qiu *et al*. [39] also confirms that patients with high family resilience tend to have better psychological resilience to cope with dialysis. More importantly, the chain mediation pathway revealed a unique potential pathway through which sleep quality affects family resilience. CBP treatment typically lasts 12 to 24 hours per session and often requires long-term or even lifelong maintenance, imposing a substantial caregiving burden on family members. When patients experience poor sleep quality—manifested as frequent nocturnal awakenings, restlessness, or nighttime discomfort—family carers often face disrupted sleep themselves, leading to cumulative physical and emotional exhaustion. Over time, this bidirectional sleep disturbance may erode the family’s capacity for effective communication, problem-solving, and emotional support, which are core components of family resilience. Furthermore, patients’ sleep disorders may increase daytime irritability and reduce their engagement in family activities, further straining family interactions and diminishing the family system’s ability to cope with treatment-related stressors. This “sleep-driven erosion” of family functioning represents a distinct pathway particularly salient in the CBP population, given the intensive and recurrent nature of the treatment. In addition, this study found that sleep quality was negatively associated with family resilience, which may be because patients’ sleep disorders increase the psychological burden on family carers and weaken their supportive capacity, creating a chain reaction of “depression → poor sleep → reduced family resilience → reduced adherence”. This explains why the chain-mediated pathway is statistically significant even though the effect size is only 5.06%—reflecting the complexity of the multifactorial interaction. However, given the cross-sectional design of this study, we can only demonstrate statistical indirect association patterns rather than causal mediation or definitive mechanisms. The observed chain pathways should be interpreted as potential relationships rather than confirmed causal effects.

From a clinical perspective, the effect-size hierarchy revealed in this study provides a basis for prioritizing interventions. The direct effect of depressive mood on treatment adherence accounted for the largest proportion (58.26%), followed by the indirect pathways through sleep quality (23.36%) and family resilience (13.32%), with the chain-mediated pathway contributing 5.06%. Accordingly, we propose a stepped-care intervention plan. Firstly, routine screening for depressive symptoms should be implemented at CBP initiation, with timely psychological or pharmacological support. Secondly, for patients with poor sleep quality, interventions such as optimizing treatment schedules to minimize nocturnal disruption and providing sleep hygiene education should be prioritized, as improving sleep may prevent subsequent erosion of family resilience. Thirdly, for patients with persistent sleep disturbances, family-based approaches—including carer education on sleep-related burden, communication training and respite care—should be activated. This stepwise model, aligned with the effect-size hierarchy, offers a practical framework for breaking the chain pathway of “depression → poor sleep → reduced family resilience → reduced adherence”.

This study has several limitations. Firstly, the cross-sectional design precludes causal inferences regarding the relationships among depressive mood, sleep quality, family resilience, and treatment adherence. Future longitudinal studies are needed to verify the dynamic pathways observed. Secondly, the single-centre sample may limit generalizability. Moreover, key clinical confounders—including illness severity, cognitive status, cumulative treatment exposure, and psychosocial burden—were not adjusted for, and these factors may independently influence the proposed mediating variables. Thirdly, this study did not distinguish between different CBP modalities (e.g., CVVH vs. CVVHD), which may differ in treatment duration, hemodynamic stability, and patient tolerance, potentially affecting patients’ mood and compliance; future studies with larger samples should perform subgroup analyses. Fourthly, the DTAS, originally developed for maintenance haemodialysis patients with end-stage renal disease, has not been formally validated for acute or acute-on-chronic CBP populations. Although the scale showed acceptable internal consistency (Cronbach’s α = 0.82) in our sample, this supports reliability for group-level comparisons but does not fully address construct validity. Therefore, our adherence findings should be interpreted as exploratory, and future studies should prioritize developing or validating CBPspecific adherence instruments. Finally, the exclusion of patients with severe comorbidities or recent major negative life events may have resulted in a relatively healthier sample, potentially limiting the generalizability to more complex patient populations. Despite these limitations, this study provides novel insights into the chain mediating effects of sleep quality and family resilience in patients with CBP.

## Conclusions

This study analysed data from 195 patients receiving CBP treatment, showing that depressive mood is directly and negatively associated with treatment compliance. Moreover, sleep quality and family resilience show significant chain indirect associations in this process, accounting for 41.74% of the total association. We recommend that mood screening, sleep management and family support be incorporated into an integrated intervention programme: early identification and intervention of depression, implementation of individualized sleep therapy, and enhancement of family resilience through family education and resource linkage. This integrated strategy is expected to break the vicious circle of “depression → sleep disorder → family function decline → reduced adherence”, and ultimately improve patients’ prognosis and quality of life.

## Availability of Data and Materials

All data included in this study can be obtained by contacting the corresponding author if needed.
